# Antimalaria Effect of the Ethanolic Stem Bark Extracts of *Ficus platyphylla* Del

**DOI:** 10.1155/2011/618209

**Published:** 2011-11-24

**Authors:** Isma'il Shittu, Amlabu Emmanuel, Andrew Jonathan Nok

**Affiliations:** ^1^Department of Biochemistry, Ahmadu Bello University, Zaria, Nigeria; ^2^Department of Biochemistry, Kogi State University, P.M.B 1008 Anyigba, Nigeria

## Abstract

The antimalarial effect of the ethanolic stem bark extract of *Ficus platyphylla* Del was evaluated against *Plasmodium berghei* infection in mice. Nontreated, experimental control mice died of fulminant parasitemia from day 7 to 9 post-infection but mice treated with the extract at 300 mg/kg showed markedly reduced parasitaemia bouts of 43.50% and a mean survival time of 28 days postinfection. The plant extract prevented a drastic reduction in PCV showing its efficacy in ameliorating anaemic conditions in *Plasmodium berghei*-infected mice. Histological examination of liver tissues of treated and untreated mice further supports the antimalaria potential of this plant. This observation validates the traditional use of this plant for the treatment of malaria.

## 1. Introduction

Malaria, a disease caused by Plasmodium species, is one of the oldest and greatest health challenges affecting 40% of the world's population [1]. It affects 300–500 million people and kills 1.5–2.7 million people annually [2].

World Health Organization has estimated mortality rate of children less than 5 years in Nigeria to be 729 children per 100,000 children (Government in action report).

The disease is a major obstacle to economic advancement of many developing and tropical nations posing people to poverty and disease.

One of the areas for the search of new antimalarials is the use of traditionally claimed antimalarial plant from the African flora [3, 4].

*Ficus platyphylla *Del belongs to the family Moraceae; its common name is broad leaf fig. The stem bark of *Ficus platyphylla* is used traditionally to treat malaria in Africa [5] and in treating tuberculosis [6].

The extracts of *Ficus platyphylla* have also been reported to inhibit gastrointestinal motility [7]. It has been reported to possess analgesic [8], anti-inflammatory, and anticonceptive activities [9].

Previous reports have shown that *Ficus sycomorus* L possesses antimalaria potentials against *Plasmodium falciparum in vitro *[10].

Medicinal plants have been the focus for the search of new antimalaria drugs in various parts of the world [11] and the present global situation indicates a recent resurgence in the severity of malaria, due to the resistance of malaria parasites to mainstay antimalaria drugs [12]. Hence, there is need to intensify research in the development of new, cheap and effective antimalaria drugs from medicinal plants.

We began to investigate in rodent malaria models the antimalarial activity of *Ficus platyphylla* used experientially as ingredients in the traditional remedies for malaria in Africa and have evaluated the *in vivo *antimalarial effect of the ethanolic stem bark extract of *Ficus platyphylla* Del in *Plasmodium berghei*-infected mice.

## 2. Materials and Methods

### 2.1. Plant Material

*Ficus platyphylla* stem barks were collected from the vicinity of Bakori dam at Bakori local government, Katsina state. The plant was identified at the Department of Biological Sciences, Ahmadu Bello University, Zaria, Nigeria, and a voucher specimen number 7230 has been deposited at the Departmental herbarium.

### 2.2. Extract Preparation

The stem bark of *Ficus platyphylla* was dried for two weeks, and the dried plant material was ground into fine powder. Eighty (80) grams of the powder was soaked in ethanol (250 mL) and placed in an orbital shaker for 48 hours. The ethanolic extract was filtered with a muslin cloth, and the filtrate was evaporated to dryness in a temperature-regulated water bath preset at 40°C. The recovered extracts from the ethanolic extract (filtrate) were weighed and formulated in dextrose saline to give the required dose.

### 2.3. BALB/c Mice

Mice were obtained from the Faculty of Pharmaceutical Sciences, Ahmadu Bello University, Zaria, Nigeria and were maintained on food and water ad libitum at the animal house situated at the Department of Biochemistry, Ahmadu Bello University, Zaria, Nigeria, and the guide for the care and use of laboratory Animals, 1996 of the Institute of Laboratory Animal Research (ILAR) Commission on life Science, National Research Council was duly followed.

### 2.4. Malaria Parasite

The malaria parasite (*Plasmodium berghei*) was obtained from the National Institute of Medical Research, Lagos in Nigeria.

### 2.5. Parasite Inoculation

Parasitized erythrocyte was obtained from donor mice by cardiac plexus puncture and was diluted with trisodium citrate. Mice were inoculated intraperitoneally with 0.2 mL blood suspension containing 10^6^–10^7^parasitized erythrocytes on day 0.

Thirty (30) of the infected mice with parasitemia of 9–12% were randomly divided into six groups of five (5) mice per group. The extract and standard drug administration was done for four days postinfection.

### 2.6. Treatment Regimen

Day 4 postinfection, treatment commenced at the establishment of parasitemia for four days.

Group 1 was infected but not treated.
Group 2 was treated with 10 mg/kg of the standard antimalaria drug (artemether).
Group 3 was treated with 100 mg/kg of the ethanolic extract.
Group 4 was treated with 200 mg/kg of the ethanolic extract.
Group 5 was treated with 300 mg/kg of the ethanolic extract.
Group 6 was not infected but administered dextrose saline alone in which the extract was formulated.

### 2.7. Parasitemia Determination

Thin smears of blood films were made from the peripheral blood collected from the tail of each mouse during and after infection [13, 14]. The smears made on microscopic slides were fixed in methanol and stained with Giemsa, pH 7.2. The numbers of parasitized erythrocytes in each 10–50 fields were counted thrice. The average was computed to give the parasitemia of each mouse.

### 2.8. Determination of Hematocrit Packed Cell Volume

The packed cell volume (PCV) was determined to predict the effectiveness of the ethanolic extract [15]. Blood was drawn from the tail of the mice in the different groups. Duplicate and triplicate determinations of the haematocrit packed cell volume were done to determine the relative volume of blood occupied by erythrocytes using the expression:

(1) $$\begin{array}{cc} & \text{Packed Cell Volume}\\ & \;=\frac{\text{Volume of erythrocytes in a given volume of blood}}{\text{Total blood volume}}. \end{array}$$

### 2.9. Histopathological Analysis

Representative samples of the livers of mice were excised on day 9 postinfection from the untreated experimental control, naïve control group of mice, and the 300 mg/kg treated group of mice and were fixed in 10% formalin, stained with haemotoxylin and eosin, and microscopic examination for tissue degeneration was carried out.

## 3. Results

The results of this study indicated that ethanolic stem bark extracts of *Ficus platyphylla *Del displayed antimalarial activity in a dose dependent fashion when compared to the infected, untreated experimental controls. 

**Figure 1:**
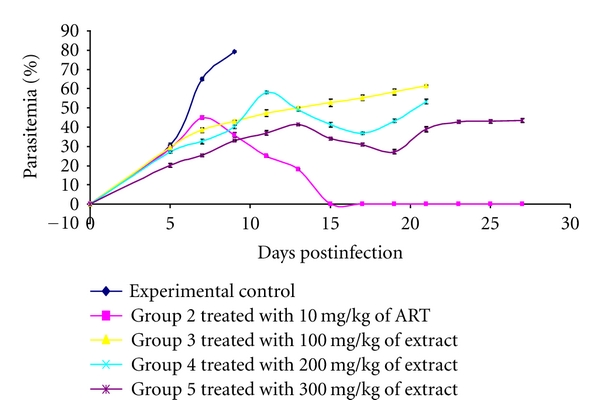
Parasitemia profiles of mice infected with *Plasmodium berghei *and treated with various doses of stem bark ethanolic extract of *Ficus platyphylla* Del.

The 4-day suppressive test carried out at the different doses of the test extract indicated that at 100 mg/kg, 200 mg/kg, and 300 mg/kg, there was a dose-dependent reduction in parasite load when compared to the infected, untreated experimental controls (Figure 1).

**Figure 2:**
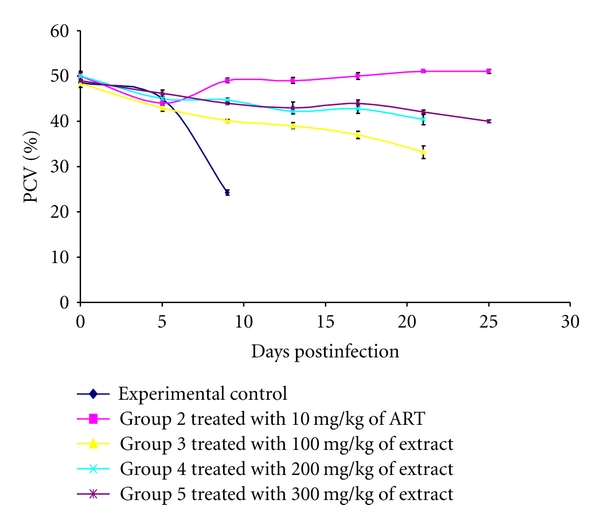
Packed cell volume (PCV) profiles for mice infected with *Plasmodium berghei *and treated with various doses of stem bark ethanolic extract of *Ficus platyphylla *Del.

Also, mice in the infected, untreated experimental control group showed drastic reduction in packed cell volume (PCV) values as the infection progresses. This was prevented significantly (*P* < 0.05) in mice treated with the ethanolic extract at 200 mg/kg and 300 mg/kg dose on comparison with PCV values obtained from infected, untreated controls (Figure 2).

**Figure 3:**
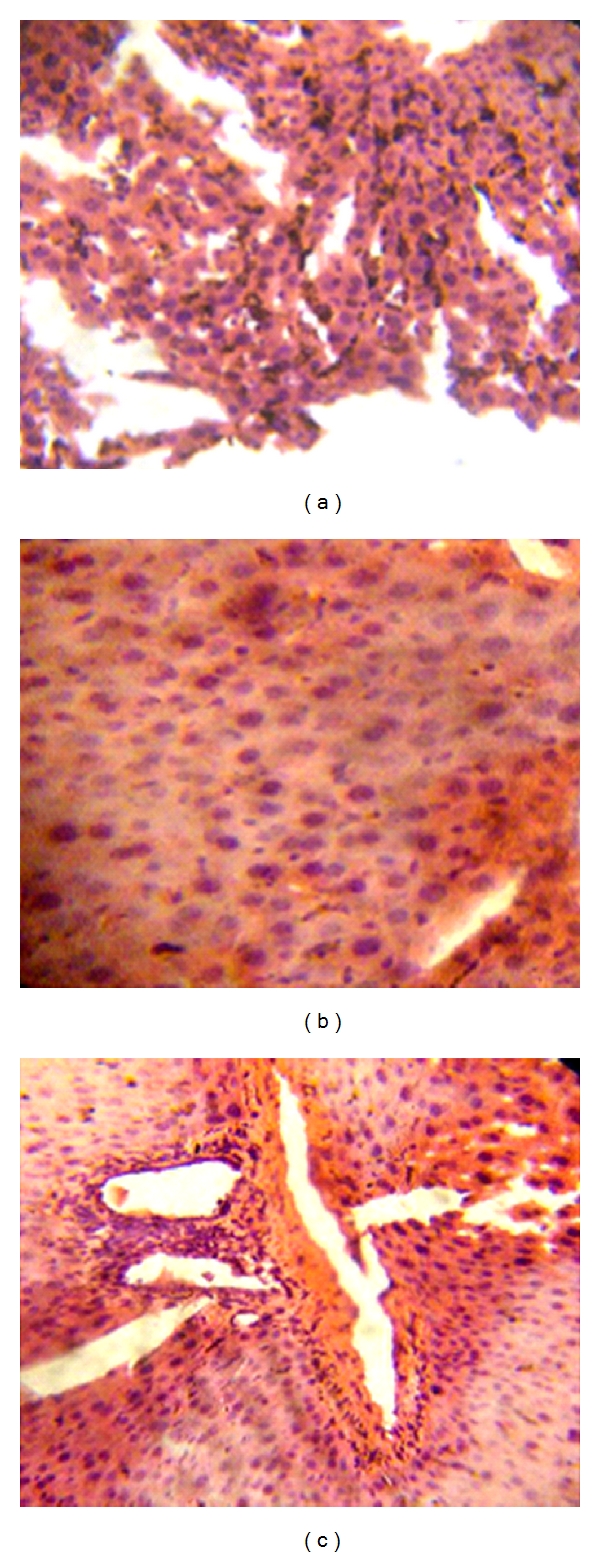
(a) Liver photomicrograph of *Plasmodium berghei*-infected mice showing dilated hepatic sinusoids congested with hypertrophied Küpffer's cells-laden malaria pigment and parasitized red blood cells (Haematoxylin and Eosin stain, ×200), (b) Liver photomicrograph of *Plamodium berghei*-infected mice treated with 300 mg/kg dose of *Ficus platyphylla* Del. Micrograph shows progressive clearance of Küpffer's cells-laden malaria pigment (Haematoxylin and Eosin stain, ×200), (c) Liver photomicrograph of naive experimental control mice showing normal lobular architecture of the liver (Haematoxylin and Eosin stain, ×200).

Haematoxylin and eosin stain of liver tissues obtained from mice in the infected, untreated experimental control group shows dilated hepatic sinusoids congested with hypertrophied, Küpffer's cells-laden malaria pigment and parasitized red blood cells (Figure 3(a)). While photomicrograph of the group of mice treated with the 300 mg/kg dose of the extract revealed progressive clearance of Küpffer's cells-laden malaria pigment and normal lobular architecture. (Figure 3(b)), also, photomicrograph of the naive experimental control group of mice showed normal lobular architecture of the liver (Figure 3(c)).

## 4. Discussion

Prediction of the efficacy of several conventional antimalarial drugs such as chloroquine, halofantrine, mefloquine, and more recently artemisinin derivatives has been identified [14] and validated in rodent models even though primate models provide a better prediction of efficacy than rodent models.

A mean group parasitemia level which is less than or equal to 90% of mock-treated control animals usually indicates that the test compound is active in standard screening studies [14].

Therefore, it is clear from the results of this study that the treatment of infected mice with the ethanolic stem bark extracts of *Ficus platyphylla *Del reduced the erythrocytic stage development of *P. berghei.*

When a standard antimalarial drug is used in treatment of mice infected with *P. berghe*i, it suppresses parasitemia to undetectable levels [16] which is in agreement with the antimalaria effect of artemether in this study.

The plant extract did not eradicate parasites completely, but it exhibited a marked reduction in multiplication of parasites during treatment, indicating that the extract may have a direct action on the parasites. Also, a major drawback in the use of medicinal plants is the partial loss of activity against parasites when administered *in vivo* which may be due to lack of uptake of the extracts to physiologically active levels [17].

Some bioactive compounds have been reported to be present in the Ficus species such as tannins, saponins, flavonoids, steroids, anthraquinone glycosides, and reducing sugars [18, 19], and several plant constituents like, flavonoids, tannins, quinonoid, xanthene, polyphenols, and terpenoids have been reported to possess protein-binding and enzyme-inhibiting properties [20, 21].

Based on the foregoing, we suggest that a likely mechanism of action of this plant may be the inhibition of key pathogenic enzymes of the malaria parasite since these bio-compounds are known to interfere with enzyme systems.

The ethanolic extract prevented a drastic reduction in PCV values in infected mice, an index of anemia [22] when compared to infected, untreated experimental controls showing its efficacy in ameliorating anemic conditions in infection. This was consistent with the marked decrease in parasite load observed in the course of infection in the groups of mice treated with the 300 mg/kg and 200 mg/kg doses of the ethanolic extract.

Results obtained from the histopathological studies further support the efficacy of the plant extract on *Plasmodium berghei* infection since tissue biopsy may be a valuable tool to establish diagnosis when other diagnostic methods are inconclusive considering the fact that histopathological alterations due to malarial infection in the liver are specific [23].

The liver was congested with black pigmentation as a result of haemoglobin metabolism by the parasite which leads to the production of hemozoin which consists of iron and protein moiety. The iron porphyrin complex has been reported to be phagocytized and processed by the macrophages in the tissues resulting in the dark pigmentations on the liver [24].

It can be concluded that the stem bark extracts of *Ficus platyphylla *possess antimalaria effects and justify the traditional usage of the plant as antimalaria remedy.

Further work on the present study is ongoing in the area of bioassay-guided fractionation in order to isolate and characterize the bioactive component(s) of the plant extract.
